# Xeroderma pigmentosa associated with spindle cell carcinoma of lung: a case report

**DOI:** 10.1097/MS9.0000000000003261

**Published:** 2025-04-04

**Authors:** Bibek Shrestha, Grishma Kandel, Dhiraj Adhikari, Sudip Bastakoti, Abhinash Mishra

**Affiliations:** a Maharajgunj Medical Campus, Tribhuvan University, Institute of Medicine, Kathmandu Nepal; b Department of Internal Medicine, Tribhuvan University Teaching Hospital, Kathmandu Nepal

**Keywords:** DNA damage, spindle cell carcinoma, ultraviolet rays, xeroderma pigmentosum

## Abstract

**Introduction and importance:**

Xeroderma pigmentosum is a rare autosomal recessive genetic disorder characterized by defective nucleotide excision repair, leading to extreme sensitivity to ultraviolet radiation, skin pigmentation changes, and a heightened risk of malignancies. Neurological symptoms, such as seizures and cognitive decline, are observed in 20% of cases. While xeroderma pigmentosa is commonly associated with skin cancers, systemic malignancies like lung carcinoma are exceedingly rare.

**Case presentation:**

A 22-year-old male with a history of xeroderma pigmentosa presented with a six-month history of non-productive cough, hemoptysis, intermittent fever, and episodic jerky movements in his upper limb. Examination revealed hypo- and hyperpigmented macules, cachexia, cervical lymphadenopathy, and diminished air entry in the left lung. Blood tests indicated leukocytosis, elevated ESR, and abnormal electrolyte levels. Imaging confirmed a left lower lobe lung mass, and biopsy revealed spindle cell carcinoma with p53 positivity on immunohistochemistry.

**Clinical discussion:**

Spindle cell carcinoma is a rare and aggressive subtype of non-small-cell lung cancer, comprising 0.2–0.3% of pulmonary malignancies. Its association with XP is notable, as defective DNA repair mechanisms in xeroderma pigmentosa predispose patients to malignancies driven by p53 mutations. This case emphasizes the need for vigilance for systemic malignancies in XP patients.

**Conclusion:**

This is the first reported case of xeroderma pigmentosa associated with spindle cell carcinoma of the lung and focal seizures. It underscores the importance of early recognition and comprehensive surveillance for rare malignancies in XP patients, given their elevated cancer risk. The patient opted for palliative care and was symptomatically managed.

## Introduction

Xeroderma pigmentosum (XP) is an uncommon genetic condition that is characterized by sensitivity to ultraviolet rays, ultimately leading to severe sunburn reactions and increased risk of skin cancer^[1]^. Patients require stringent photoprotection measures, including sunscreen application and protective clothing^[2]^. The clinical manifestations include hypo and hyperpigmented macules, premature aging, sunburns, blistering, and increased incidence of different malignancies in the skin. It also affects the eye and central nervous system^[3]^. Patients involved in the eye can experience progressive blindness^[4]^. A complex relationship exists between XP and other malignancies, which is poorly understood^[2]^. Priya and Janaki (2017) reported rare cases of carcinoma of the esophagus associated with XP^[5]^. Though susceptible to different cancers, few cases have been reported till now due to rare nature^[6]^. There is an association with neurological symptoms in 20 % of cases^[4]^.

**HIGHLIGHTS**

- The case underscores the role of defective DNA repair mechanisms in XP, particularly mutations involving the p53 gene, which contribute to the development of systemic malignancies.
- Beyond its cutaneous manifestations, XP patients are at risk for systemic malignancies and neurological complications, necessitating a multidisciplinary approach to management.
- The case emphasizes the importance of regular cancer surveillance and early intervention in XP patients to detect rare malignancies and manage comorbid conditions effectively.

Herein, we present the case of a 22-year-old male diagnosed with XP who developed spindle cell carcinoma and experienced focal seizures. This case report is the first documented case associating XP, spindle cell carcinoma, and focal seizures. This case has been reported following the SCARE Guideline^[7]^

## Case presentation

A 22-year-old male nonalcoholic and nonsmoker with a known history of XP diagnosed at 6 months of age presented to the hospital with chief complaints of a non-productive, intermittent cough for 6 months, accompanied by episodes of hemoptysis, intermittent fever, and shortness of breath of the same duration. He developed a non-productive, intermittent cough associated with shortness of breath and left-sided chest pain 6 months back. Additionally, he reported intermittent fever, with the highest recorded temperature of 102°F, accompanied by chills and rigors. He also had jerky movements on the upper limb at night for a few minutes, mainly during sleep. He had 4–5 jerky movements till now. His medical history was notable for cicatricial ectropion with corneal opacity and neovascularization, for which he underwent eye surgery ten years ago.

On examination, the patient appeared ill and had cachexia but had no pallor, icterus, lymphadenopathy, cyanosis, clubbing, or signs of dehydration. His vital signs were a heart rate of 80 beats per minute, a respiratory rate of 18 breaths per minute, a blood pressure of 110/70 mmHg, a temperature of 98°F, and an SpO_2_ of 98% on the right arm. He had level II right cervical lymphadenopathy, which was palpable (1X1 cm) and tender. His skin exhibited generalized hypo- and hyperpigmented macules all over the body. Chest examination revealed crackles and decreased air entry on the left side. Cardiovascular examination showed normal heart sounds (S1 and S2), and the central nervous system examination was unremarkable.

**Figure 1.**
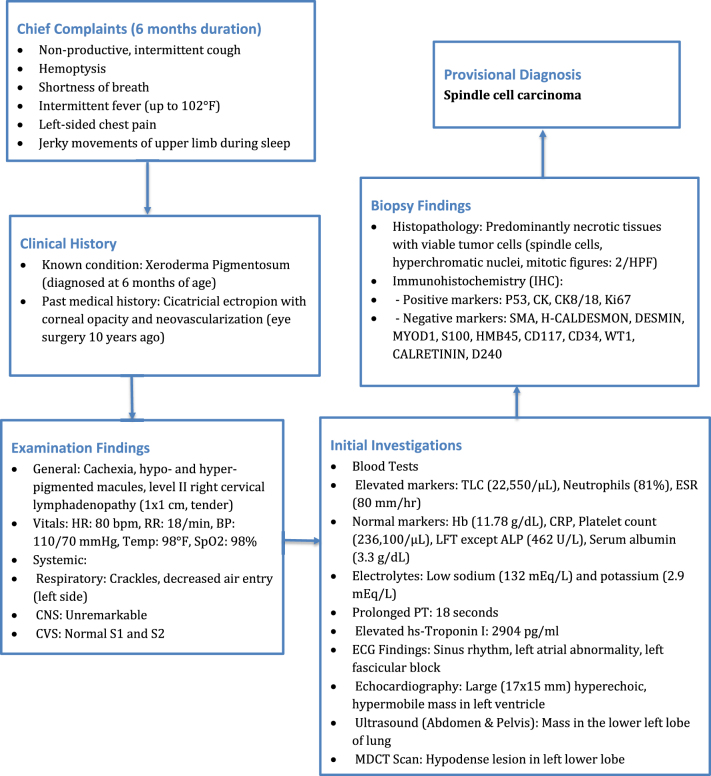
Flowchart: diagnostic workup for a 22-year-old male with xeroderma pigmentosum.

Potential cardiac and pulmonary pathologies were considered for further diagnostic workup, including infective endocarditis, mediastinal mass, and lung or intrathoracic malignancy. These conditions were evaluated as part of the differential diagnosis. Blood investigations were sent, and hemoglobin was found to be 11.78 g/dL (normal range: 13–18 g/dL), and the total leukocyte count was elevated at 22 550 cells/µL (normal range: 4000–11 000 cells/µL), with a differential count showing neutrophils at 81% (normal range: 45–75%) and lymphocytes at 8% (normal range: 25–45%). The erythrocyte sedimentation rate (ESR) was significantly elevated at 80 mm/hr (normal range: 0–12 mm/hr), but C-reactive protein was in the normal range. The platelet count was 236 100 cells/µL (normal range: 150 000–400 000 cells/µL). Prothrombin time (PT) was prolonged at 18 seconds (normal range: 11–14 seconds). Serum sodium was slightly low at 132 mEq/L (normal range: 135–145 mEq/L), and serum potassium was notably reduced at 2.9 mEq/L (normal range: 3.5–5.2 mEq/L). The blood sugar level was in the normal range and was 77 mg/dl (normal range: 74–130 mg/dl); hsTroponin I was significantly high and was 2904 pg/ml (standard: less than 12 is negative). The patient’s liver function test results were as follows: total protein was 6.4 g/dL (reference range: 6.4–8.2 g/dL), total bilirubin was 0.8 mg/dL (reference range: 0–1.1 mg/dL), direct bilirubin was 0.4 mg/dL (reference range: 0.0–0.4 mg/dL), alanine transaminase (ALT) was 34 U/L (reference range: 0–50 U/L), aspartate aminotransferase (AST) was 43 U/L (reference range: 0–45 U/L), alkaline phosphatase was elevated at 462 U/L (reference range: 40–140 U/L), and serum albumin was slightly low at 3.3 g/dL (reference range: 3.8–4.9 g/dL). Electrocardiography was sent, and he was reported to have sinus rhythm along with left atrial abnormality and left fascicular block (Fig. 1). Echocardiography revealed multiple hyperechoic, hypermobile large masses (17 x 15 mm) attached to the left ventricle suggestive of embolized tumor mass. (Fig. 1) The significance of the left atrial abnormality and the left fascicular block on ECG, along with the echocardiographic findings suggestive of an embolized tumour mass, has been further elaborated in relation to the patient’s overall clinical picture.

**Figure 2.**
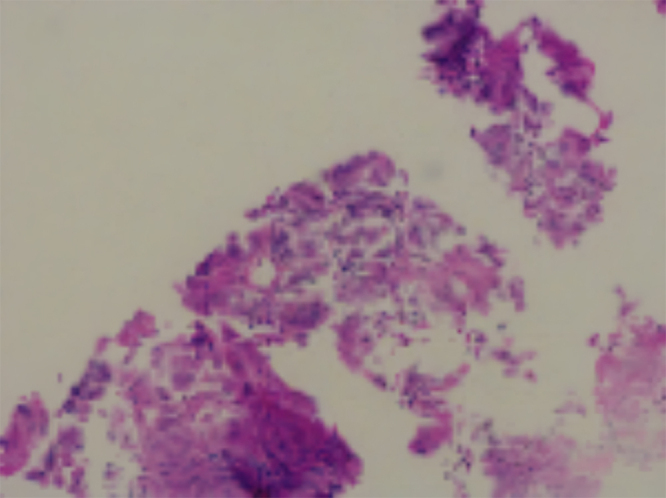
Trucut biopsy section.

**Figure 3.**
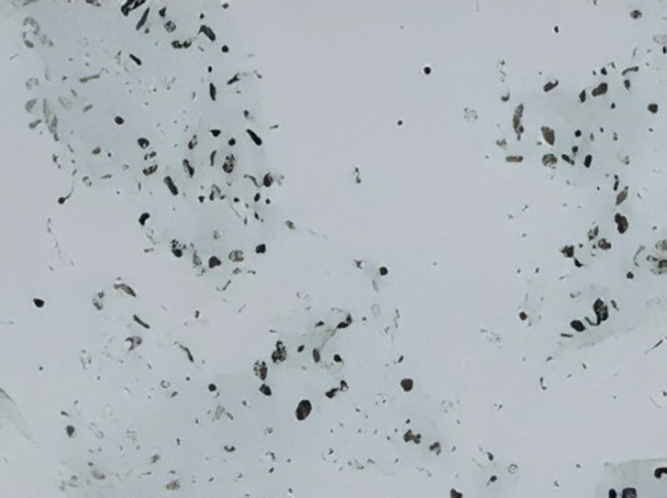
Ki67 (BH360).

**Figure 4.**
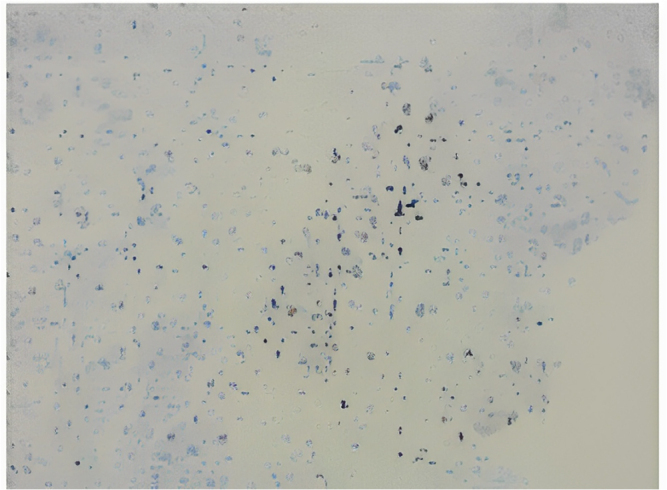
P53 positive.

After ruling out other pathologies based on the patient’s history, physical examination, and initial investigations, intrathoracic malignancy became the leading suspicion. Ultrasound of the abdomen and pelvis was performed, revealing a mass in the left lower lobe of the lung. To further confirm the diagnosis, a multi-detector computed tomography (MDCT) scan was conducted, which demonstrated a mild heterogeneously enhancing hypodense lesion in the left lower lobe, suggestive of a malignant lung mass. Additionally, the MDCT identified metastatic lymph nodes and mild pleural effusion. Ultrasound-guided Tru-cut needle biopsy was sent from the left lung mass, and it revealed multiple cores of necrotic tissues composed of small areas of the viable tumor with a sheet of fascicles of spindle cells with a diagnosis of spindle cell tumors. The biopsy section showed multiple cores of a neoplasm which is composed of predominantly necrotic tissues with pockets of small viable areas of tumors and tumors composed of sheets and fascicles of monomorphic spindle cells, with oval to spindled hyperchromatic nucleus with mitotic figure 2/high power field (Fig. 2). To further confirm, an immunohistochemistry study was conducted, and the tumor cells tested positive for p53, with a rare cell showing positivity for cytokeratin (CK), cytokeratin 8/18 (CK8/18), and Ki67 (Fig. 3 and Fig. 4). The tumor cells were negative for smooth muscle actin (SMA), H-caldesmon, desmin, MYOD1, S100, and HMB45, excluding smooth muscle differentiation and rhabdomyosarcoma, respectively. Additionally, the tumor cells were negative for the signal transducer and activator of transcription 6 (STAT6), CD117, and CD34, which excludes a solitary fibrous tumor and an extra-intestinal gastrointestinal stromal tumor (GIST), respectively. The tumor cells were also negative for SALL4 and octamer-binding transcription factor 3/4 (OCT3/4), excluding germ cell tumors, and negative for Wilms’ tumor 1 (WT1), calretinin, and D240, excluding malignant mesothelioma. Furthermore, the tumor cells were negative for mouse double minute 2 (MDM2) and cyclin-dependent kinase 4 (CDK4), excluding liposarcoma; however, BRG1, BRCA-associated protein 1 (BAP1), integrase interactor 1 (INI1), and histone H3 lysine 27 trimethylation (H3K27ME3) were retained. The CD markers were further evaluated where CD99, CD21, and CD23 were negative. The diagnosis was primarily based on histopathological examination and immunohistochemistry (IHC) findings. The tumor showed spindle-shaped cells arranged in fascicles, with positivity for p53, cytokeratin (CK), and CK8/18, which are characteristic of SCC. In contrast, other sarcomatoid carcinomas, such as pleomorphic carcinoma or carcinosarcoma, often exhibit a mix of epithelial and mesenchymal components with different IHC profiles. Additionally, markers for smooth muscle differentiation (SMA, desmin), neural tumors (S100), and mesothelial differentiation (WT1, calretinin) were negative, ruling out alternative diagnoses. These findings confirmed the diagnosis of spindle cell carcinoma and distinguished it from other sarcomatoid malignancies. To manage the patient’s skin condition, strict sun avoidance was recommended. Black goggles were prescribed for ocular protection to mitigate the risk of UV-ray-induced damage. Additionally, following a neurological consultation, the patient was diagnosed with focal seizures and initiated on carbamazepine 200 mg, to be taken once daily. The patient chose palliative care due to financial constraint of his family. To prevent pulmonary edema in lung run due to lung cancer, furosemide and spironolactone twice a day for 7 days were given. For chest pain and preventing further hypertension, metoprolol 25 mg twice a day was advised. Rivaroxaban 20 mg once a day per oral was advised to reduce thrombosis risk in the future. Upon follow-up at 1 month, 2 months, and 6 months, the patient showed improvement, with his symptoms resolving completely, and he did not experience any epileptic seizures.

## Discussion

XP is an unusual autosomal recessive genetic skin condition caused by nucleotide excision repair mutations or DNA damage. Severe photosensitivity in ultraviolet rays, skin pigmentary changes, malignant tumor formation, and, on rare occasions, progressive neurologic deterioration distinguish the illness. Patients may also exhibit oral, ophthalmologic, and neurologic signs of the condition^[8]^. Patients with XP have congenital abnormalities in the deoxyribonucleic acid (DNA) damage repair system, which makes their skin very vulnerable to UV exposure^[6]^. Though rare, XP can be associated with lung malignancy. Lung cancer in XP patients is caused by the carcinogen benzo(a)pyrene binding to their DNA, which is caused by defective DNA damage repair mechanisms^[9]^. In XP patients, defective DNA repair leads to the accumulation of UV-induced p53 mutations, promoting genomic instability and uncontrolled cell proliferation. While commonly seen in skin cancers, these mutations may also drive the development of spindle cell carcinoma in the lung by disrupting tumor suppressor pathways. The loss of p53 function contributes to the aggressive nature of malignancy, linking XP-associated DNA repair defects to rare systemic cancers^[10]^. When associated with lung cancer, common symptoms include cough, wheezing, dyspnea, and hemoptysis^[11]^. In this case, we present a case of a 22-year-old male with a known history of XP who came with a non-productive cough associated with hemoptysis, intermittent fever, and shortness of breath. XP is known to cause progressive neurological decline in approximately 20% of cases due to defective nucleotide excision repair (NER), leading to cumulative DNA damage in neurons. This can result in cortical atrophy, cerebellar dysfunction, and peripheral neuropathy, which may contribute to seizures. However, neurodegeneration in XP is typically slowly progressive, and seizures in such cases are more commonly seen in later stages of the disease, often accompanied by cognitive impairment and other neurological deficits, which were not prominently noted in this patient^[12]^. Blood parameters have emerged as significant prognostic factors in lung cancer patients. There can be deranged lymphocyte counts, platelet counts, albumin levels, and other inflammatory markers^[13]^. Metastasis can further derange other blood markers, including thyroid and liver function tests, depending upon the organ it invades. In this, the total leukocyte count, neutrophils, the erythrocyte sedimentation rate (ESR), prothrombin time, and alkaline phosphatase were found to be increased.

Non-small-cell lung cancer has various histological subtypes and treatment approaches^[14]^. One of the non-small cell lung cancers is spindle cell carcinoma. This is the first case report associated with spindle cell carcinoma and XP. Spindle cell carcinoma is an aggressive and unusual form of lung cancer, representing only 0.2–0.3% of primary pulmonary cancers^[15]^. In a study of lung tumors, spindle cell carcinoma was among the rarer, with squamous cell carcinoma being the most common. It can metastasize to the brain, causing neurological symptoms such as vertical one-and-a-half syndrome. Endobronchial biopsy findings reveal that squamous cell carcinoma is the most common centrally arising lung tumor, followed by small cell carcinoma and adenocarcinoma, with spindle cell carcinoma being relatively uncommon^[15]^. Endoscopic ultrasound-guided biopsy has emerged as a valuable alternative to conventional biopsy methods for various tissues. Endoscopic and ultrasound-guided biopsies are effective techniques for diagnosing lung cancer and mediastinal lymphadenopathy^[16]^. In this case, the patient underwent an endoscopic ultrasound-guided trust biopsy of left lung mass, and it revealed multiple cores of necrotic tissues composed of small areas of the viable tumor with a sheet of fascicles of spindle cells with a diagnosis of spindle cell tumors. The immunohistochemical (IHC) profile of spindle cell carcinoma is critical for accurate diagnosis and differentiation from other lung tumors. Key markers such as p53, cytokeratin’s (CK), and Ki-67 are commonly assessed in the evaluation. In the context of spindle cell carcinoma, p53 mutations are frequently observed, contributing to the aggressive nature of this carcinoma. Studies have shown that the aberrant expression of p53 is associated with a poor prognosis in various lung cancers^[17,18]^. Specifically, p53 overexpression can indicate a mutation that leads to uncontrolled cell proliferation, further complicating the clinical management of the disease^[18]^. Cytokeratin is a group of proteins that are key components of the cytoskeleton in epithelial cells. In spindle cell carcinoma of the lungs, the expression of cytokeratin’s, particularly CK5/6 and CK8, is often evaluated through IHC. Research indicates a significant proportion of expressed pan-cytokeratin markers, which aids in distinguishing these tumours from other spindle cell lesions^[19]^. High Ki67, which is a proliferation marker, is often found to be associated with aggressive behaviour and poor clinical outcome^[17]^. In this case, immunohistochemistry was positive for P53, and a rare cell positive for CK, CK8/18, and Ki67.

Multi-detector computed tomography (MDCT) has shown promise in lung cancer screening and diagnosis. MDCT and its post-processing techniques can accurately detect primary trachea and central bronchus tumors, providing crucial information for surgical treatment^[20]^. Recent studies have highlighted the significance of electrocardiogram (ECG) changes in non-small-cell lung cancer (NSCLC) patients. Specific ECG abnormalities, such as an increased ventricular rate, QRS voltage decrease, ST-segment depression, and new atrial fibrillation, were associated with higher mortality within three months in NSCLC patients compared to controls^[21]^. In this, the patient had sinus rhythm, left atrial abnormality, and left fascicular block with average QRS voltage and ST segment. The main treatments for lung cancer include surgery, chemotherapy, radiotherapy, and immunotherapy, often used in combination with multimodality therapy^[22]^. While survival is a primary goal, patients and caregivers also prioritize quality of life and functionality when defining treatment success^[23]^. Treatment decisions should consider factors such as expected performance status, toxicity, and hospitalization rates. For spindle cell lung cancer, first-line treatment typically involves platinum-based chemotherapy, with the addition of immunotherapy agents like atezolizumab or durvalumab in some cases^[24]^. However, due to expensive chemotherapy and immunotherapy, the patient was under palliative care with different medications. Current evidence suggests that cancer surveillance in XP should be individualized based on the patient’s clinical presentation and risk factors. Generally, dermatologic examinations for early detection of skin malignancies are recommended every 3 to 6 months^[25]^. Given the increased risk of systemic malignancies, such as the rare spindle cell carcinoma of the lung reported in our case, additional screening modalities may be considered. For internal malignancies, annual imaging studies such as chest X-rays, low-dose computed tomography (LDCT) for lung cancer screening, and abdominal ultrasound may be warranted in high-risk cases. Whole-body MRI can be considered for broader surveillance in patients with neurological symptoms or suspected malignancies. Regular neurological and ophthalmological evaluations are also recommended, given the association of XP with neurodegeneration and ocular malignancies^[26]^.

The limitation of the research is that this study could not track the effectiveness of cancer therapy, which includes platinum-based chemotherapy and immunotherapy, due to the patient chosen palliative care; however, this case report has highlighted the critical association between xeroderma pigmentosum and spindle cell carcinoma and tracked the diagnostic and treatment approach.

## Conclusion

This case highlights the rare association of xeroderma pigmentosum (XP) with spindle cell carcinoma of the lung, emphasizing the heightened malignancy risk due to defective DNA repair mechanisms, particularly p53 mutations. Given the predisposition of XP patients to both cutaneous and systemic cancers, this report underscores the necessity of vigilant malignancy surveillance and early detection strategies. A multidisciplinary approach, incorporating dermatologic, oncologic, and neurologic evaluations, is essential for optimizing patient care and outcomes.

## Data Availability

The datasets used and/or analyzed during the current study are available from the corresponding author upon reasonable request.
